# Effectiveness of mindfulness training on pregnancy stress and the hypothalamic–pituitary–adrenal axis in women in China: A multicenter randomized controlled trial

**DOI:** 10.3389/fpsyg.2023.1073494

**Published:** 2023-03-02

**Authors:** Shulei Wang, Chen Zhang, Mengyun Sun, Daming Zhang, Ying Luo, Kairu Liang, Tao Xu, XiaoPing Pan, Ruimin Zheng, Fangfang Shangguan, Jia Wang

**Affiliations:** ^1^National Center for Women and Children’s Health, China CDC, Beijing, China; ^2^Yantai Center for Disease Control and Prevention, Yantai, Shandong, China; ^3^Fengtai Mental Health Center, Beijing, China; ^4^School of Psychology, Capital Normal University, Beijing, China; ^5^Shanxi Maternal and Child Health Hospital, Taiyuan, Shanxi, China; ^6^Shandong Maternal and Child Health Hospital, Jinan, Shandong, China; ^7^Sichuan Maternal and Child Health Hospital, Chengdu, Sichuan, China; ^8^Shandong Provincial Hospital, Jinan, Shandong, China

**Keywords:** mindfulness, stress, HPA axis, randomized controlled trial, pregnant women, multicenter

## Abstract

**Introduction:**

In the past two decades, mindfulness-based intervention programs have gradually become popular.Many studies have confirmed that these programs can effectively alleviate prenatal stress and negative emotion.The mindfulness-based stress-buffering hypothesis suggests that mindfulness training can induce changes in the levels of the cortisol secreted by the HPA axis, thereby reducing stress susceptibility. However, to date, only a few high-quality evidence-based medical studies have analyzed the effect of the mindfulness-based intervention in a maternal population.Thus, this study investigated the effects of a mindfulness-based psychosomatic intervention on pregnancy stress and the HYPERLINK “javascript:;” hypothalamic-pituitary-adrenal (HPA) axis of pregnant Chinese women.

**Methods:**

Women experiencing first-time pregnancy (n = 117) were randomly allocated to the intervention group or parallel active control group, and data were collected at baseline and post-intervention periods. The participants completed questionnaires regarding mindfulness and pregnancy stress. Saliva samples was collected at the time of waking up, and 30, 45, and 60 min after waking up for analyzing the salivary cortisol levels. We analyzed differences between the two groups and changes within the same group before and after the intervention.

**Results and discussion:**

A total of 95 participants completed the trial. Compared with the parallel active control group, the intervention group exhibited lower levels of stress after the intervention (P = 0.047). For HPA-axis-related indicators after the intervention, Delta value (P = 0.01) and AUCM value (P = 0.031) of the intervention group were significantly higher than that of the control group. Mindfulness-based interventions effectively reduced the level of pregnancy stress and adjusted the HPA axis function in pregnant women in China.

**Clinical Trial Registration:**

https://www.chictr.org.cn, identifier ChiCTR 2000033149.

## 1. Introduction

Stress refers to the adaptive physiological changes, which occur when an individual is challenged by internal or external stressors, to improve their chances of survival (Elder, 1998). The transition to motherhood is a unique phase that frequently necessitates immediate and significant changes in a woman’s daily life, including her thinking and behavior. This shift in lifestyle may cause difficulties and stress for some women (Di Florio et al., 2013). A study of 1,522 pregnant women in the United States found that 78 and 6% of pregnant women experienced low to moderate and high levels of stress, respectively, during pregnancy (Woods et al., 2010). In a cross-sectional study, Mei et al. (2021) showed that the detection rate of stress in pregnant women in China was 69.39%. Stress during pregnancy has been associated with various health problems in women, including increased anxiety and postpartum depressive symptoms (Yim et al., 2015; Dunkel Schetter et al., 2016). In addition, stress during pregnancy increases negative maternal emotions, the risk of panic disorder, drug use, postpartum marital conflict, and multiple medical comorbidities (Sapolsky, 1998; Yim et al., 2015; Dunkel Schetter et al., 2016). Austin and Leader (2000) found that negative maternal emotions directly affect the fetus by altering the expression of related genes, causing changes in placental glucocorticoid signaling and, thus, increasing fetal exposure to cortisol. Austin et al. (2005) showed that the fetuses of pregnant women experiencing high stress levels had decreased fetal heart rate-movement coupling, which may indicate slower central nervous system development and more birth complications. Pregnancy stress has also been linked to adverse birth outcomes, such as preterm birth and low birthweight (Van den Bergh et al., 2020).

The activation of the hypothalamic-pituitary-adrenal (HPA) axis provides the body with the energy needed to cope with stress. However, over activation of the HPA axis can cause over-alertness in individuals (Lazarus and Folkman, 1984), and chronic dysregulation in biological stress-related systems is associated with adverse health outcomes (Chrousos, 2009). The end-product of the HPA axis is cortisol, which is released by the adrenal glands and is considered to be one of the main markers of the biological stress response (Fries et al., 2009; Caulfield and Cavigelli, 2020). Serum free cortisol levels peak at approximately 20–45 min after waking, and subsequently decline throughout the day. The dynamic pattern of cortisol, known as the cortisol arousal response (CAR), and indicators related to the CAR can be used to measure the physiological stress response function of the HPA axis (Pruessner et al., 1997; Chojnowska et al., 2021). A dramatic change in HPA axis regulation and cortisol secretion occurs during pregnancy, with cortisol levels rising throughout pregnancy and returning to pre-pregnancy levels after childbirth. This plays a key role in fetal organ development (Mastorakos and Ilias, 2003). Cortisol is associated with a heightened risk of stress-related health complications during pregnancy and postpartum (Zijlmans et al., 2015; Hodyl et al., 2017). Moreover, a blunted or flat CAR is correlated with an increased risk of postpartum depression (Scheyer and Urizar, 2016). Furthermore, flatter diurnal cortisol slopes (i.e., a smaller decrease in cortisol levels throughout the day) during pregnancy have been linked to increased anxiety and impaired sleep quality in mothers, as well as low birthweight in infants (Kivlighan et al., 2008; Bublitz et al., 2018).

Effective intervention measures are necessary to improve the physiological stress response function of the HPA axis and reduce stress during pregnancy. Pregnant women, especially first-time mothers, require organized antenatal education and preparation for birth. In the past two decades, mindfulness-based intervention programs have gradually become popular as a method of helping people improve their well-being. A mindfulness-based childbirth and parenting (MBCP) program (Hughes et al., 2009; Bardacke, 2012) was developed for pregnant women in the United States and adopted by Bardacke from the widely known and effective mindfulness-based stress reduction (MBSR) program developed by Kabat-Zinn (2003). The MBCP aims to teach pregnant women and their partners mindfulness skills to manage negative emotions and stress, during pregnancy and encourage sensitive parenting styles (Duncan and Bardacke, 2010; Duncan et al., 2017). Studies in several countries have confirmed that these MBCP programs can effectively alleviate prenatal stress and negative emotions (Vieten and Astin, 2008; Hofmann et al., 2010; Khoury et al., 2013). The mindfulness-based stress-buffering hypothesis suggests that mindfulness training can induce changes in the levels of the cortisol secreted by the HPA axis, thereby reducing stress susceptibility (Creswell et al., 2014). Some reviews suggest that mindfulness training may ameliorate stress-related diseases by decreasing the HPA axis response to acute stress (Creswell et al., 2019). However, to date, only a few high-quality evidence-based medical studies have analyzed the effect of the MBCP intervention in a maternal population, and no studies have explored the effect of the MBCP intervention on pregnancy stress and the HPA axis of Chinese pregnant women.

China has a unique traditional, cultural, and social background in maternal health care. In 2016, our team introduced the MBCP to China. We conducted a preliminary survey with Chinese pregnant women to determine the demand for the course and discovered that several pregnant women did not accept the traditional 9-week MBCP course because it was deemed too long. We increased the MBCP program’s compatibility with Chinese culture and social context to meet the needs of pregnant women in China, thus, increasing the likelihood of maternal participation in the course. Our team modified the traditional MBCP curriculum in China (Bardacke, 2019) by changing the 9-week course to a “2-day on-site and 21-day online” curriculum model and simplifying parts of the curriculum. The present study explored the efficacy of the simplified version of the MBCP course in reducing pregnancy stress and improving HPA axis function in Chinese pregnant women.

## 2. Materials and methods

### 2.1. Study design and sample size

In this randomized controlled trial, we compared the efficacy of a simplified version of the MBCP intervention program in reducing stress and regulating salivary cortisol levels in pregnant women with that of a control group. Using statistical power analysis, we calculated the required sample size. The perceived stress scale (PSS) score was considered as a reference. The results of a previous MBCP study (Pan et al., 2019a,b) showed mean (and standard deviation [SD]) value of 11.64 (SD = 6.13) and 14.29 (SD = 5.23) on the PSS scale for the intervention group and control groups; and a statistical power of 0.90 was used to reject a null effect at a 0.05 level of significance. The minimum sample size was estimated to be 44 for each group (88 total). The target sample size was set as 110 participants after considering a possible attrition rate of 20%.

We generated random grouping sequences using SAS version 9.1 for Windows and assigned participants to the control or the intervention group (1:1 ratio) based on their time of enrollment. In our study, the participants were blinded and were not aware about the grouping.

### 2.2. Participants

**Figure 1:**
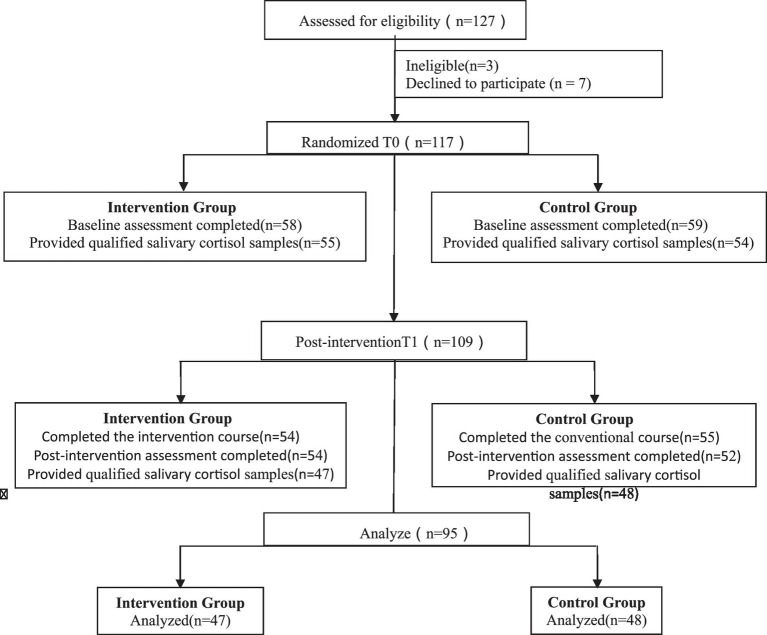
CONSORT diagram. Passage of participants through each trial stage.

This multicenter randomized controlled study was conducted from August 2021 to April 2022 at three hospitals: Shanxi Maternal and Child Health Hospital, Shandong Maternal and Child Health Hospital, and Shandong Provincial Hospital. The pregnant women were recruited from the three hospitals. Women who met the following criteria were invited to participate: singleton pregnancy between 20 and 32 weeks, able to communicate adequately in Chinese, had set up a registry at the target hospital, and planned to undergo prenatal examination, in-hospital delivery, and postnatal review at the hospital. Furthermore, the women had to have a minimum academic qualification of high school education. Otherwise eligible pregnant women were excluded if they had a history of psychiatric disturbances, epilepsy, multiple abortions, or premature birth, with serious pregnancy complications or diseases, such as severe pregnancy-induced hypertension or heart disease, which may prevent them from participating in the study. We initially selected 127 women to participate in the study (Figure 1).

### 2.3. Interventions

The intervention group received a 2-day on-site simplified version of the MBCP course over a weekend. The course lasted 6 h per day, including 3 h in the morning and 3 h in the afternoon, with a total on-site intervention time of 12 h. Two MBCP teachers, with extensive teaching experience, conducted the on-site course in small groups of approximately 30 people. The on-site course consisted primarily of raisin meditation, breathing awareness, body scan, mindful yoga and meditation, labor pain cognitive education, and pain management with ice holding exercises (Table 1). Within 21 days of a completion of the on-site course, the participants in the intervention group participated in the 21-day online course *via* the WeChat applet with recorded audio, which lasted for 5–35 min per day. The online course included formal practice, such as mindful breathing, body scan, mindful yoga, and 3-min breathing space, as well as informal practice, such as mindful eating, mindful tooth brushing, mindful face washing, and other daily mindfulness practices. At the same time, the control group received an online pregnancy and childbirth education course with a recorded video *via* the WeChat applet for 21 days, which lasted approximately 5–10 min per day. This course covered physical and psychological knowledge related to pregnancy and self-care skills during pregnancy and postpartum.

**Table 1:** The main content of the 2-day on-site course.

Day 1	Introduction to mindfulness and introduction of the teacher and the participants. Practice: mindfully eating a raisin; awareness of breathing meditation; mindful yoga;
	Body scan meditation; mindful walking.
	Requesting; sharing among participants.
	Psycho-education: physiology of childbirth and pain from a body–mind perspective.
	Explanation of parenting knowledge: the neonatal sleep and diet rules.
	Practice: pain meditations using ice and a variety of pain-coping strategies;
	requesting, sharing among participants.
Day 2	Review of the course; encouragement to continue practicing mindfulness.

Additionally, the participants in the intervention group received the same regular pregnancy and childbirth health education as the control group. While, the participants in the control group only received the regular pregnancy and childbirth health education for 21 days. We encouraged the partners of the participants in the intervention and control group to accompany and participate in the courses. Using the WeChat platform, we assisted and guided the two groups of participants in completing the relevant study and exercises. We arranged for physicians to respond to their pregnancy-and childbirth-related questions *via* the WeChat platform.

### 2.4. Data collection and measures

At the time of recruitment, all participants completed the demographic information questionnaire. In addition, we collected the Five Facet Mindfulness Questionnaire (FFMQ), PSS, and saliva samples from the two groups within 1 week before and after the intervention. Participants collected saliva samples by themselves at home at four time points in the morning: at awaking (AC_0min_), and 30 min (AC_30min_), 45 min (AC_45min_), and 60 min (AC_60min_) after awaking. At the post-intervention evaluation, participants in the intervention group completed the client satisfaction questionnaire (CSQ), designed by our team, to measure satisfaction with the course on a 10-point scale (1–10, with higher scores indicating greater satisfaction with the course). The participants received and completed the questionnaires in the presence of the investigators. The subjects read and filled the questionnaires by themselves. After the questionnaires were collected, we arranged two researchers to double input the questionnaire information into the database using the EpiData3.1 software.

#### 2.4.1. Five facet mindfulness questionnaire

Mindfulness was assessed using the FFMQ. The scale is based on factor analysis and consists of 39 items, yielding subscale scores that measure five elements of mindfulness: observing, describing, acting with awareness, being non-judgmental of inner experience, and being non-reactive to inner experience. Responses were scored using a 5-point Likert-type scale, with 1 representing “never or very rarely true” and 5 representing “very often or always true” (Baer et al., 2006). In our study, the Cronbach’s alpha ranged from 0.79 to 0.88.

#### 2.4.2. Perceived stress scale

The PSS was used to assess stress. Chen created the scale in 1983 and revised it in 1989 to measure the psychological stress level of women during pregnancy. The scale contains 30 items and 4 subscales: stress from ensuring the health and safety of the mother and child (PSS1), validating a parent’s role (PSS2), altering body structure and function (PSS3), and other factors (worried about taking care of the baby, worried about not being able to maintain a good spousal relationship after having a baby, and worried about not being able to provide good living conditions for the baby). For each question, 0 to 4 points are awarded; the higher the score, the greater is the pregnancy stress. The reliability coefficient of the table containing the total quantity is 0.90. The coefficients of internal consistency for the three subscales ranged from 0.79 to 0.89 (Cohen et al., 1983; Chen et al., 1989). In our study, the Cronbach’s alpha ranged from 0.82 to 0.88.

#### 2.4.3. Salivary cortisol level

To control diurnal hormonal changes, participants were asked to avoid caffeine, alcohol, or engage in aerobic exercise the day before collecting the saliva samples. They were given a saliva collection kit containing four commercially available saliva collection tubes, ice packs, thermal insulation bags, and other tools for collecting and transporting saliva samples at low temperatures. They were also given a collection log to record the dates and times of saliva collection. At enrollment, the participants were individually instructed on how to collect saliva. If necessary, the participants could practice collecting saliva in the presence of researchers, who would answer any pertinent questions on-site to improve protocol adherence. The participants collected saliva samples on their own using the saliva collection tubes at four time points on each collection day (immediately upon waking up, and 30, 45, and 60 min after waking). We asked all the participants to store each tube of saliva specimen in a 4°C refrigerator immediately after collection. On the day of collection, the participants themselves brought the saliva samples to the hospital using ice packs and thermal insulation bags, and handed them to the researchers. Subsequently, the researchers stored the specimens in a refrigerator at −80°C. After collecting all the specimens, they were transported, using dry ice and a bio-safety transport box, to the laboratory for centralized testing. After the saliva was thawed at room temperature, the collected saliva was centrifuged at 3000 rpm for 15 min, and the subnatant was collected for biochemical analysis. The salivary cortisol level was quantified using an ezymelinked immunosorbent assay (ELISA) kit (Cat. Number: SLV-2930, DRG, Germany). Centralized testing was performed within 20 days.

### 2.5. Ethical considerations

The study protocol was approved by the Ethics Review Committee of the National Center for Women and Children (Chinese Center for Disease Control and Prevention, Beijing, China; approval no. FY2020-10). Before recruitment, we explained our study’s purpose, significance, benefits, and potential risks in detail to every pregnant woman interested in participating. In addition, we explained to the participants what they needed to do in our study and follow-up to gain their cooperation and understanding. The participants provided written informed consent. In addition, to keep the data confidential, all the data collected were anonymous and prohibited for use outside of our study. We informed the participants that they had the right to withdraw from participation at any time during the study period, without consequences.

### 2.6. Data analysis

SPSS version 24.0 for Windows was used for data analysis. Demographic characteristics are presented as mean (standard deviation [SD]) for measurement data and as frequency counts (percentages) for categorical variables. Chi-square and Fisher’s exact test were used to compare the demographic variables of the two groups (education level, marital status, and family income). *t*-test was used to analyze rank scores and measurement data, including scale baselines and cortisol-related indicators. We compared the differences in questionnaire scores and cortisol-related indicators between the two groups using analysis of covariance (ANCOVA) and the generalized estimate equation (GEE) before and after the intervention, respectively.

Three cortisol-related indicators were computed: Delta, which measures the acute rise in cortisol typically seen after waking in the morning (Dedovic and Ngiam, 2015); the area under the curve with respect to the ground (AUC_G_), which measures the overall amount of cortisol secreted within 1 h after awakening (Pruessner et al., 2003); and the area under the curve with respect to increase (AUC_M_), which measures the increase in cortisol within 1 h of waking relative to the minimum of four time points (AC_min_) (Grossi et al., 2005). All the scores were calculated and used as indices of the HPA function.


$$\begin{array}{l} Delta=\left(AC_{30\min}-AC_{0\min}\right)÷AC_{0\min}\times 100\\ AUC_{G}=\left(AC_{0\min}+AC_{30\min}\right)\times 30/2+\left(AC_{30\min}+AC_{45\min}\right)\\ \times 15/2+\left(AC_{45\min}+AC_{60\min}\right)\times 15/2\\ AUC_{M}=AUC_{G}-AC_{\min}\times 60 \end{array}$$

## 3. Results

In total, 127 participants were interviewed. We eliminated the research participants with premature birth, stillbirth, and other cases of premature termination of pregnancy during the intervention process. We also excluded participants who did not complete the course (for the intervention group: participants who were absent from the on-site courses or attended fewer than 16 online courses; for the control group: participants who attended fewer than 16 online regular health education sessions). Moreover, we eliminated the cortisol data due to delay of the first sample collection by more than 5 min after awakening, an insufficient amount of saliva collected at any of the four time points, and outlier cortisol values (>3 SD from the mean). Among the 127 participants, 3 were eliminated because they did not meet the criteria for inclusion, 7 pregnant women withdrew from participation prior to the intervention, and 22 participants were excluded from further analysis because of follow-up failure or failure to provide satisfactory saliva samples. Of the 22 participants excluded from further analysis, 11 belonged to the intervention group and 11 belonged to the control group; the attrition rate was 18.8%. Finally, 95 participants were included in the data analysis, including 47 and 48 in the intervention and control group, respectively (Figure 1).

The differences between the intervention (*n* = 47) and control groups (*n* = 48) were not statistically significant for age, the infant’s gestational age, body weight, education level, census register, marital status, household income, parity, pregnancy method, and pregnancy complications. We also compared the general information between the participants who did (*n* = 95) and did not (*n* = 22) complete the entire experiment and found no statistical differences (*p* > 0.10). We compared the baseline data of the psychological questionnaires and cortisol levels prior to intervention between the two groups and found a statistically significant difference between the two groups in the dimension of ensuring maternal and child health and safety of PSS1 (*p* < 0.05), but no significant differences in cortisol levels and the other questionnaires (Table 2). At the post-intervention evaluation, 93.62% of the intervention group participants scored 8 or higher on the CSQ questionnaire, indicating that they were satisfied with the course.

**Table 2:** Comparison of general information and the baseline data between the two groups [ $\bar{x}\pm s,$ *n*(%)].

Characteristics	Intervention group (47)	Control group (48)	Statistics	*P*
Age (M ± SD)	30.43 ± 2.96	30.42 ± 3.02	0.01	0.99^a^
Gestational age of infant (M ± SD)	24.17 ± 4.23	25.71 ± 3.53	−1.93	0.16^a^
Body weight(M ± SD)	67.50 ± 11.44	65.07 ± 13.2	0.96	0.34^a^
Level of education			0.70	0.4^b^
Junior college or below	12	16		
University or above	35	32		
Marital status			<0.001	1^c^
Married	46	47		
Not married	1	1		
Income			0.84	0.66^b^
Less than ¥100,000	10	9		
¥100,000–¥200,000	28	26		
More than ¥200,000	9	13		
Parity			0.44	0.51^b^
No prior births	37	35		
1 or more prior births	10	13		
Pregnancy way			0.001	1^c^
Pregnancy by nature	44	45		
Pregnancy by medicine	3	3		
Pregnancy complications			0.15	0.74^c^
No	42	44		
Yes	5	4		
Waking time [hour:minutes (minutes)]	7:10 (±0:11)	7:36(±0:12)	1.22	0.12^a^
FFMQ	124.13 ± 7.81	126.13 ± 9.65	−1.11	0.27^a^
FFMQ-observing	23.52 ± 4.59	23.29 ± 4.44	0.24	0.81^a^
FFMQ-describing	27.47 ± 4.52	27.19 ± 4.62	0.31	0.76^a^
FFMQ-acting with awareness	29.45 ± 3.74	30.85 ± 4.07	−1.75	0.08^a^
FFMQ-non-judgmental	24.23 ± 4.53	24.38 ± 3.86	−0.17	0.87^a^
FFMQ-non-reactive	19.46 ± 2.66	20.42 ± 3.20	−1.59	0.12^a^
PSS	21.72 ± 10.70	17.92 ± 10.09	1.78	0.08^a^
PSS1	6.51 ± 4.36	6.09 ± 4.89	0.44	0.66^a^
PSS2	8.81 ± 5.00	6.70 ± 3.15	2.46	0.02^a*^
PSS3	4.17 ± 2.70	3.33 ± 2.81	1.48	0.14^a^
PSS-others	2.23 ± 1.36	1.87 ± 1.70	1.15	0.25^a^
AC				
AC_0min_	10.98 ± 4.27	11.23 ± 4.19	−0.29	0.77^a^
AC_30min_	16.85 ± 7.46	19.45 ± 7.43	−1.70	0.09^a^
AC_45min_	14.38 ± 5.78	16.66 ± 6.05	−1.88	0.06^a^
AC_60min_	13.67 ± 6.42	14.54 ± 5.10	−0.73	0.47^a^
Delta	64.51 ± 79.05	91.53 ± 91.43	−1.54	0.13^a^
AUC_M_	272.14 ± 183.98	352.51 ± 209.84	−1.98	0.05^a^
AUC_G_	862.06 ± 314.52	964.96 ± 303.88	−1.62	0.11^a^

^a^*t*-test; ^b^Chi-square test, X^2^; ^c^Fisher’s exact test; *p < 0.05. FFMQ, the five facet mindfulness questionnaire; PSS, the perceived stress scale; PSS1, stress from ensuring the health and safety of the mother and child; PSS2, stress from validating a parent’s role; PSS3, stress from altering body structure and body function; PSS-others, stress from the others; AC, awaking cortisol level; AC_0min_, cortisol level at awaking; AC_30min_, cortisol level at 30 min after awaking; AC_45min_, cortisol level at 45 min after awaking; AC_60min_, cortisol level at 60 min after awaking; Delta, the acute rise in cortisol after waking in the morning; AUC_M_, area under the curve with respect to increase, measures the increase in cortisol within 1 h of waking relative to minimum of four time points; AUC_G_, area under the curve with respect to ground, measures the overall amount of cortisol secreted within 1 h after awakening.

We compared the differences in questionnaire scores in one group before and after intervention. The result showed that the total score of the FFMQ and the dimension of observing after the intervention were significantly higher than that before the intervention (*p* = 0.001, *p* = 0.013). The other dimensions of the FFMQ also improved after the intervention compared with those before the intervention, but the difference was not statistically significant. The total score of the PSS and dimension of PPS3 in the intervention group were significantly lower after the intervention (*p* = 0.03, *p* = 0.004) than that before the intervention. The dimensions of PSS1 and PSS2 also decreased after the intervention, but the differences were not statistically significant. In the control group, the dimension of acting with awareness (FFMQ) was significantly lower after the intervention (*p* = 0.04), but there were no significant differences in the total score and other dimensions of the FFMQ before and after the intervention. The dimensions of PSS2 and PSS-others were significantly higher after the intervention than that before the intervention (*p* = 0.04, *p* = 0.01). There were no significant differences in the total score and other dimensions of the PSS before and after the intervention.

We compared the differences in questionnaire scores between the two groups before and after the intervention using an ANCOVA. The total score of the FFMQ and its five dimensions were higher in the intervention group than that in the control group after intervention; however, the difference was not statistically significant. The difference in the FFMQ total scores between the two groups was marginally significant (*p* = 0.057). Meanwhile, the total score of the PSS questionnaire in the intervention group was significantly lower than that in the control group after intervention (*p* = 0.047). The dimension “body shape and change” of the PSS was significantly lower in the intervention group than that in the control group (*p* = 0.018). The dimension “others” of the PSS was significantly lower in the intervention group than that in the control group (*p* = 0.03). Meanwhile, the dimensions “identify with parental roles” and “health and safety of mother and child” were lower in the intervention group than those in the control group, but the differences were not statistically significant (Tables 3, 4).

**Table 3:** Mean and standard deviation of questionnaire scores for both groups and comparison of the scores before and after intervention in one group ( $\bar{x}\pm s$ ).

Questionnaire	Intervention group (47)		*t*	*P*	Control group (48)		*t*	*P*
	Before intervention	After intervention			Before intervention	After intervention		
FFMQ	124.13 ± 7.81	130.65 ± 10.73	−3.37	0.001*	126.13 ± 9.65	126.49 ± 9.36	−0.19	0.85
FFMQ-observing	23.52 ± 4.59	26.14 ± 5.40	−2.54	0.013*	23.29 ± 4.44	25.00 ± 4.58	−1.88	0.06
FFMQ-describing	27.47 ± 4.52	28.61 ± 3.54	−1.36	0.18	27.19 ± 4.62	27.74 ± 3.70	0.64	0.52
FFMQ-actingwith awareness	29.45 ± 3.74	30.00 ± 4.48	−0.65	0.52	30.85 ± 4.07	29.02 ± 4.36	0.86	0.04*
FFMQ-nonjudgmental	24.23 ± 4.53	25.55 ± 3.83	−1.54	0.13	24.38 ± 3.86	25.00 ± 3.82	−0.79	0.43
FFMQ-non-reactive	19.46 ± 2.66	20.34 ± 3.34	−0.62	0.16	20.42 ± 3.20	19.73 ± 2.15	1.24	0.22
PSS	21.72 ± 10.70	16.89 ± 10.64	2.19	0.03*	17.92 ± 10.09	22.21 ± 13.38	−1.78	0.08
PSS1	6.51 ± 4.36	5.40 ± 3.93	1.30	0.20	6.09 ± 4.89	6.33 ± 4.83	−0.24	0.81
PSS2	8.81 ± 5.00	6.93 ± 3.64	2.08	0.40	6.70 ± 3.15	8.33 ± 4.54	−2.04	0.04*
PSS3	4.17 ± 2.70	2.70 ± 2.11	2.94	0.004*	3.33 ± 2.81	3.98 ± 2.81	−1.13	0.26
PSS-others	2.23 ± 1.36	1.97 ± 1.77	0.80	0.43	1.87 ± 1.70	2.79 ± 1.88	−2.52	0.01*

*p < 0.05.

**Table 4:** Results of covariance analysis.

Questionnaire	Adjusted mean and standard deviation		F	*P*	*d*	CI
	Intervention group (47)	Control group (48)				
FFMQ	130.58 ± 1.49	126.56 ± 1.46	3.70	0.057	4.01	[−0.13,8.16]
FFMQ-observing	26.14 ± 0.73	25.01 ± 0.73	1.21	0.275	1.13	[−0.92,3.18]
FFMQ-describing	28.62 ± 0.53	27.74 ± 0.53	1.39	0.241	0.88	[−0.60,2.36]
FFMQ-acting with awareness	30.05 ± 0.65	28.97 ± 0.65	1.37	0.244	1.08	[−0.75,2.92]
FFMQ-non-judgmental	25.54 ± 0.56	25.00 ± 0.55	0.48	0.492	0.54	[−1.02,2.11]
FFMQ-non-reactive	20.29 ± 0.41	19.79 ± 0.41	0.72	0.397	0.41	[−0.66,1.65]
PSS	17.00 ± 1.79	22.10 ± 1.77	4.05	0.047^*^	−5.10	[−10.14,−0.07]
PSS1	5.44 ± 0.63	6.28 ± 0.62	0.90	0.345	−0.84	[−2.60,0.92]
PSS2	6.82 ± 0.61	8.44 ± 0.61	3.43	0.067	−1.62	[−3.35,0.12]
PSS3	2.72 ± 0.37	3.97 ± 0.36	5.76	0.018^*^	−1.25	[−2.28,−0.22]
PSS-others	1.96 ± 0.27	2.80 ± 0.27	4.87	0.030^*^	−0.84	[−1.59,−0.08]

*p < 0.05.

**Figure 2:**
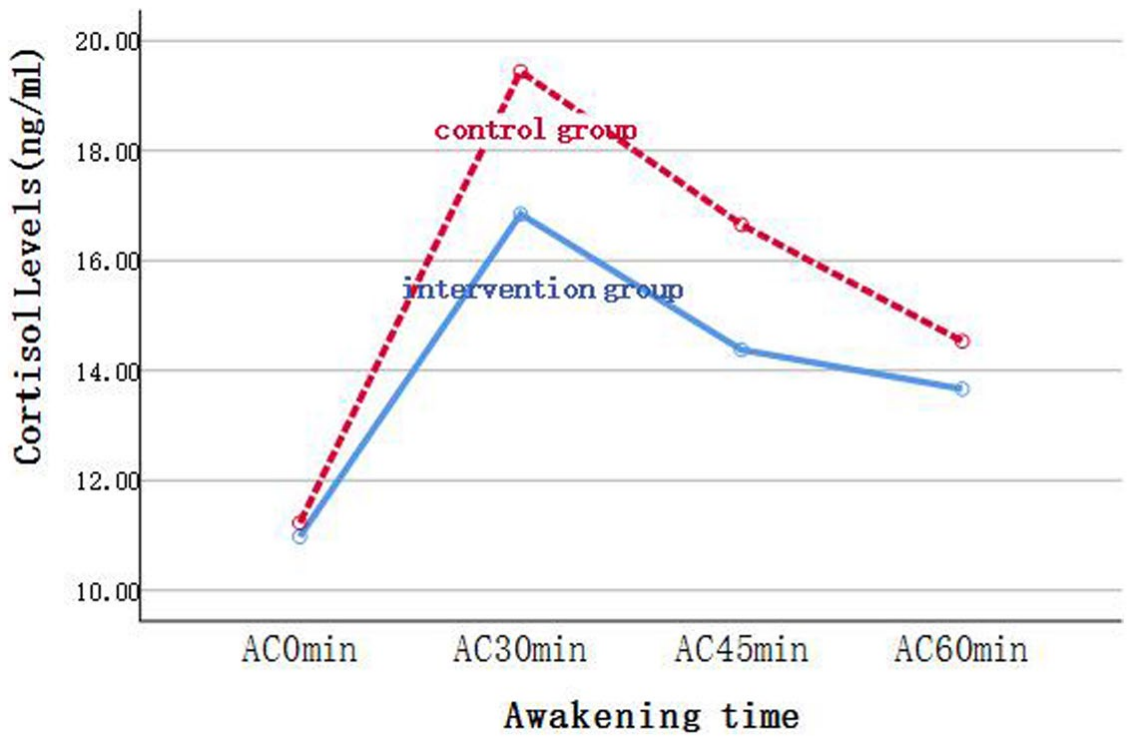
Changes of cortisol levels in saliva samples of the two groups at each time point before intervention.

**Figure 3:**
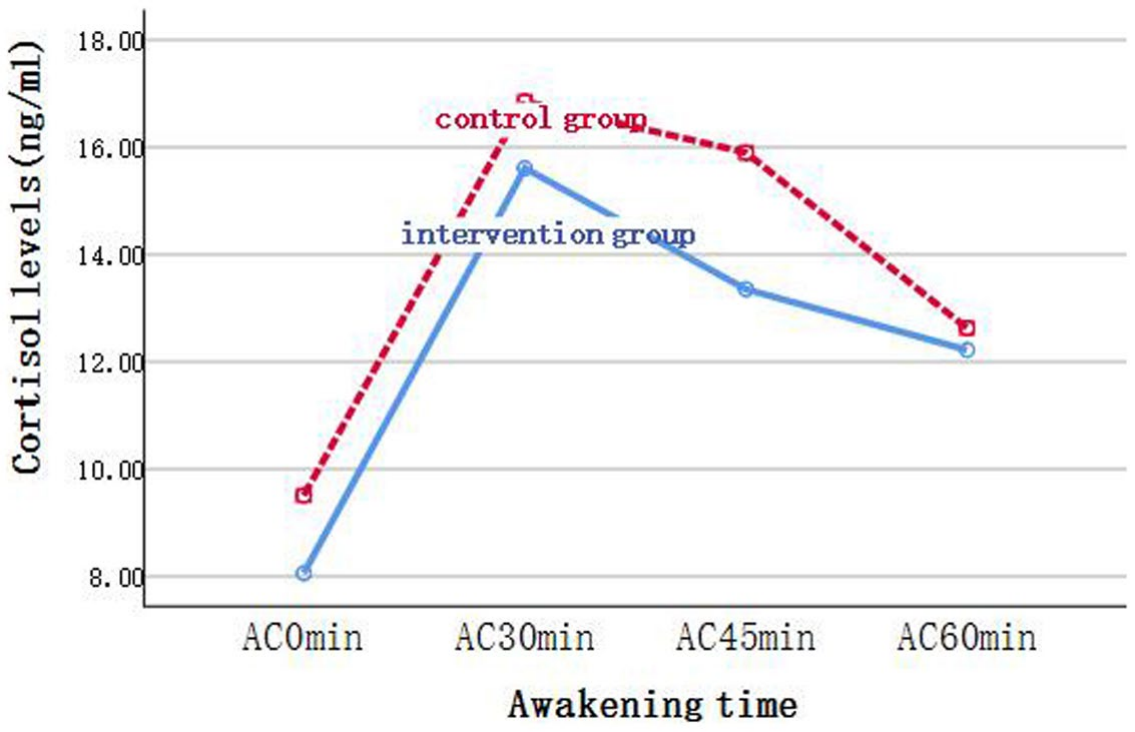
Changes of cortisol levels in saliva samples of the two groups at each time point after intervention.

**Figure 4:**
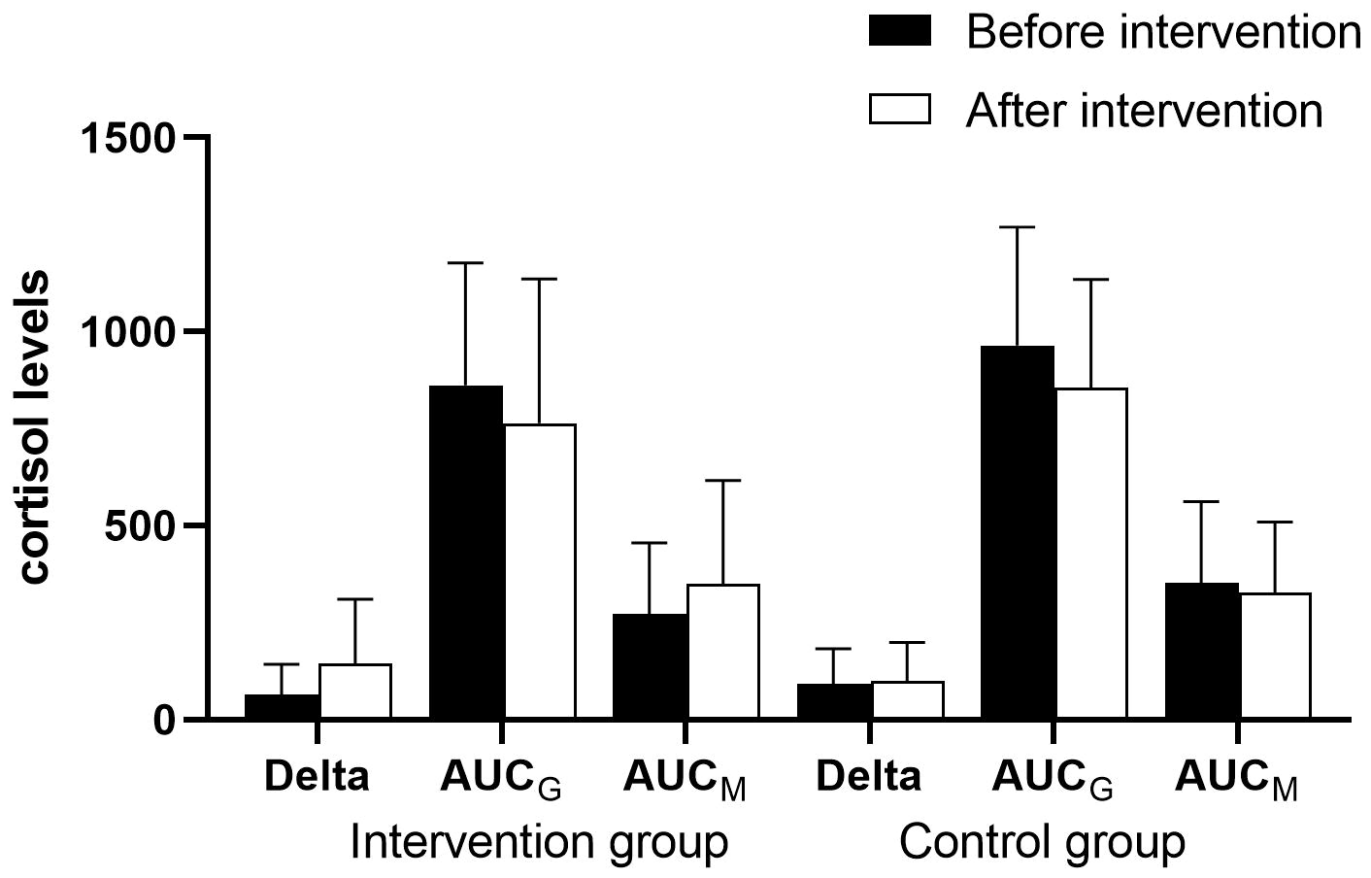
Comparison of cortisol levels (Delta, AUCG, and AUCM) of the intervention and control group before and after the intervention.

We compared the differences in cortisol levels and related indicators between the two groups before and after the intervention using the GEE. In the analysis, grouping was considered as the main effect, whereas time was considered as a covariate. For Delta, *β*_1_ = −100.35, *p* = 0.009; that is, cortisol levels in the intervention group decreased by an average of 100.35 relative to those in the control group after intervention, and the difference was statistically significant. Meanwhile, the difference in Delta before and after the intervention was not statistically significant (*β*_2_ = 8.09, *p* = 0.624). Finally, the difference in the magnitude of change between the two groups before and after the intervention was statistically significant (*β*_3_ = 73.33, *p* = 0.01). For AUC_M_, *β*_1_ = −183.43, *p* = 0.014; that is, cortisol levels in the intervention group decreased by an average of 183.43 relative to those in the control group after intervention, and the difference was statistically significant. Meanwhile, the difference in AUC_M_ before and after the intervention was not statistically significant (*β*_2_ = −25.62, *p* = 0.415). The difference in the magnitude of change between the two groups before and after the intervention was statistically significant (*β*_3_ = 103.05, *p* = 0.031). AC_0min_, AC_30min_, AC_45min_, AC_60min_, and AUC_G_ exhibited no significant difference between the two groups. For AC_0min_, AC_30min_, and AC_60min_, the differences in cortisol levels before and after the intervention were significant. After the intervention, the cortisol levels decreased by an average of 1.72, 2.59, and 1.91, respectively, compared with those before the intervention. For AUC_G_, the difference in cortisol total output before and after the intervention was significant. After the intervention, the cortisol total output decreased by an average of 109.85 compared with that before the intervention (Figures 2–4; Table 5).

**Table 5:** Results of GEE analysis.

Cortisol related indicators	Variables	*β*	SE	Wald *X*^2^	*P*
AC_0min_	(Intercept)	12.96	1.11	137.29	0
	Intervention group	0.95	1.64	0.33	0.56
	Control group	0	–	–	–
	Time	−1.72	0.66	6.75	0.009^*^
	Time*Intervention group	−1.20	1.00	1.44	0.23
	Time*Control group	0			
AC_30min_	(Intercept)	22.04	2.12	108.17	0
	Intervention group	−3.95	3.10	1.62	0.20
	Control group	0	–	–	–
	Time	−2.59	1.27	4.18	0.041^*^
	Time*Intervention group	1.35	1.96	0.48	0.49
	Time*Control group	0			
AC_45min_	(Intercept)	17.42	1.60	119.27	0
	Intervention group	−2.01	2.29	0.77	0.38
	Control group	0	–	–	–
	Time	−0.76	0.94	0.66	0.42
	Time*Intervention group	−0.27	1.45	0.03	0.85
	Time*Control group	0			
AC_60min_	(Intercept)	16.45	1.26	170.50	0
	Intervention group	−1.33	2.10	0.40	0.53
	Control group	0	–	–	–
	time	−1.91	0.68	7.83	0.005^*^
	Time*Intervention group	0.46	1.22	0.15	0.70
	Time*Control group	0			
Delta	(Intercept)	83.45	26.09	10.23	0.001
	Intervention group	−100.35	38.24	6.89	0.009^*^
	Control group	0	–	–	–
	Time	8.09	16.50	0.24	0.62
	Time*Intervention group	73.33	28.54	6.60	0.01^*^
	Time*Control group	0			
AUC_M_	(Intercept)	378.14	55.67	46.14	0
	Intervention group	−183.43	74.90	6.00	0.014^*^
	Control group	0	–	–	–
	Time	−25.62	31.45	0.66	0.42
	Time*Intervention group	103.05	47.80	4.65	0.031^*^
	Time*Control group	0			
AUC_G_	Intercept	1074.82	76.91	195.28	0
	Intervention group	−114.84	118.22	0.94	0.33
	Control group	0	–	–	–
	Time	−109.85	43.24	6.46	0.011^*^
	Time*Intervention group	11.93	72.61	0.03	0.87
	Time*Control group	0			

*p < 0.05.

## 4. Discussion

This randomized controlled trial aimed to examine the effects of the simplified version of the MBCP course on reducing pregnancy stress and regulating the CAR in pregnant Chinese women during pregnancy compared with the active control group. A total of 95 pregnant women completed the study. The demographic characteristics and baseline cortisol-related indicators were not statistically significantly different between the intervention and control group before the intervention. However, after the intervention, the level of pregnancy stress was significantly reduced, whereas the levels of Delta and AUC_M,_ were significantly increased in the intervention group compared with that in the control group.

In terms of mindfulness intervention for pregnancy stress, the conclusion of this study is consistent with the conclusions of most previous studies (Krusche et al., 2018; Warriner et al., 2018; Pan et al., 2019a,b; Lönnberg et al., 2020), but the effect was different. Warriner et al. (2018) of Oxford University conducted a single-arm study involving 86 pregnant women. In this study, the participants received a 4-week mindfulness intervention, and their stress levels were compared before and after the intervention to evaluate the intervention effect of the course. Their results showed that the stress level was 4.13 points lower after intervention. Meanwhile, in our study, the stress level of the participants in the intervention group decreased by 4.83 points, which was slightly higher than Warriner et al. (2018). However, the baseline level of pregnancy stress (19.19 ± 7.03) among pregnant women in the previous study was slightly lower than that of participants in the intervention group (21.72 ± 10.07) of our study. Participants with a higher level of stress may be more sensitive to the intervention. In addition, the study by Warrine et al. had a high rate of loss to follow-up (41.9%) and did not include a control group; these factors may lead to bias. Moreover, Krusche et al. (2018), also from Oxford University, conducted a randomized controlled study to evaluate the effect of an online mindfulness intervention course on pregnancy stress. Their study included 185 pregnant women, and the baseline level of pregnancy stress (21.65 ± 8.02) was similar to that in our study. In the intervention group, the level of pregnancy stress decreased by 4.47 points more than that in the control group. Meanwhile, in our study, the stress level of participants in the control group showed an upward trend after intervention, whereas the stress level of pregnant women in the intervention group decreased by 9.12 points more than that in the control group after intervention. However, Krusche et al. (2018) reported a high attrition rate (61.08%). Beattie et al. (2017) conducted a pilot randomized trial with 48 pregnant Australian women who were 24–28 weeks pregnant to evaluate the effects of an 8-week mindfulness intervention program on pregnancy stress and other adverse psychological conditions. In contrast to our research, this study measured stress using PSS-10. After the intervention, the reduction in pregnancy stress score in the intervention group was 24.28%, which is slightly higher than the reduction in our study (22.23%). Nevertheless, the attrition rate in their study (58.33%) was significantly higher than that in our study (18.8%). On the one hand, the 8-week intervention course with a longer class may have a greater intervention effect; on the other hand, the longer class may make it challenging for participants to adhere to it. Lönnberg et al. (2020) conducted a randomized controlled study involving 193 pregnant women with high stress levels. The participants were 15–22 weeks pregnant, and the intervention group received 8 weeks of MBCP training; the PSS was used to measure the level of stress. The baseline level of maternal stress was significantly higher in the intervention group (26.82 ± 7.76) than that in our study (21.72 ± 10.07). The results showed that the reduction in stress levels in the intervention group was 6.11 points greater than that in our study (4.83). Lonnberg selected pregnant women with a high stress level, and the effect of mindfulness training may be more pronounced in those with mood disorders. In conclusion, our intervention model is effective and has unique advantages for pregnancy stress. The attrition rate in our study is lower than most similar studies, especially the study with an 8-week intervention course model. In addition, our course may prove more effective intervention for pregnant women with high stress levels. In the future, it is necessary to further test the intervention effect of our course in a high-stress population.

After intervention, the Delta and AUC_M_ values of the intervention group were significantly higher than those of the control group, whereas there were no statistically significant differences in the cortisol level at awakening, and 30, 45, and 60 min after awakening. Moreover, the total amount of cortisol secreted within 1 h after awakening (AUC_G_) showed no significant difference between the two groups. Khoury et al. (2015) revealed that “total cortisol output” and “change in cortisol levels” were the two most important cortisol indicator components. Stalder et al. (2016) proposed that AUC_M_ was a sensitive indicator of the cortisol awakening response (CAR). Delta and AUC_M_ were both sensitive indicators of the CAR in our study. Few studies have been conducted on the effects of mindfulness intervention targeting the HPA axis in pregnant women. However, the results of this study are consistent with those of similar studies conducted on the non-pregnant population. For instance, Harris A R (2016) conducted a randomized controlled study of 64 educators and found that after mindfulness intervention, the CAR levels in the intervention group were significantly higher than those in the control group, whereas AUC_G_ levels did not differ significantly between the two groups. Meanwhile, Matousek et al. (2011) analyzed changes in CAR-related indicators before and after intervention in 33 women who underwent a mindfulness-based stress reduction (MBSR) program after breast cancer drug therapy. They discovered that CAR increased significantly after intervention. When they subdivided the sample further, they found that those with higher initial cortisol levels experienced a decline over time, whereas those with lower initial levels experienced an increase. Furthermore, a randomized controlled study of 114 police officers conducted by Grupe et al. (2021) drew different conclusions. They found that mindfulness training improved the mental health and sleep quality and reduced the CAR of police officers. In conclusion, mindfulness training may have a bidirectional effect on the CAR, which varies depending on the psychosomatic health level of the participants. In a study by Matousek et al. (2011) on cancer patients with poor physical functioning, CAR was negatively correlated with fatigue, and the patients had lower cortisol levels than healthy people (Chida and Steptoe, 2009). By increasing CAR, mindfulness training may have increased physical vitality. While the police force is dominated by young and middle-aged males who are physically fit and face more work pressure, CAR is relatively higher in the general population, and chronically high CAR levels are associated with overreaction, worry, burnout, and depression (Schlotz et al., 2004; Fries et al., 2009). A moderate reduction in cortisol levels is beneficial for police officers’ physical and mental health. O’Leary et al. (2016) explored the effect of mindfulness on cortisol through a meta-analysis. The idea of changes in cortisol flexibility is supported when interpreting inconsistencies in the results of assorted studies. Dedovic et al. (2010) showed that the flexibility of CAR reflects the coping abilities of the individual.

According to the arousal response hypothesis, CAR allows organisms to respond to the stress of the upcoming day, and increased cortisol levels in the morning may reflect an increase in energy demand (Schulz et al., 1998). Powell and Schlotz (2012) hypothesized that CAR was an adaptive anticipatory response for the upcoming day. When participants showed increased CAR, they experienced less distress in response to daily stress. Moreover, the increase in CAR in healthy pregnant women is lower than that in non-pregnant women (Clow et al., 2004; Shea et al., 2007; Entringer et al., 2010). This difference may result from physiological adaptations that protect the mother and fetus from overexposure to stress hormones (Christian, 2012). However, with the rapid increase in cortisol levels in pregnant women, the CAR of pregnant women decreases with an increase in gestational weeks (De Weerth and Buitelaar, 2005; Entringer et al., 2010). Thayer et al. (2018) studied 741 women who were not pregnant, who were in various stages of pregnancy, and had given birth. The results showed that the CAR of pregnant women decreased significantly in the second trimester. The trend of continuous weakening may have different effects on pregnant women’s physical and psychological outcomes at different stages of pregnancy. Meanwhile, Scheyer and Urizar (2016) found that a smaller arousal response was significantly associated with postpartum depressive symptoms in the first and second trimesters. Most of the pregnant women selected in this study were in the middle of pregnancy; therefore, it is beneficial to improve their CAR to protect the pregnant women and fetuses. A review suggested that reduced CAR may be associated with individual psychosomatic disorders, such as chronic pain or sleep disorders (Fries et al., 2009). Several studies have confirmed that the reduction in AUC_M_, an indicator of arousal increase, is associated with the aggravation of subclinical symptoms (Dedovic et al., 2010; Mangold et al., 2012; Dedovic and Ngiam, 2015). The increase of AUC_M_, an indicator of increased CAR, has great potential to improve pregnant women’s physical and mental health. Therefore, mindfulness training may be one of the effective ways to help pregnant women enhance their CAR and improve their physical and mental health.

Based on the dual effects of mindfulness training on psychological status and CAR levels, it is hypothesized that the underlying mechanism of mindfulness intervention is the mutual influence of psychological symptoms and physiological stress response, to improve the health level of the body (McEwen, 2004; Wintermann et al., 2016). In their review, Creswell et al. (2019) proposed the effect of the stress-buffering framework of mindfulness training on physical health. This suggests that both the peripheral physiological response and subjective experience, that is, the co-regulation of inflammation and additional physiological responses and health behaviors, are coping resources of the body. In the framework, psychological symptoms and HPA axis response are bidirectional. The present study found that mindfulness training may have simultaneous effects on mental health and the HPA axis, thereby verifying the framework’s hypothesis. According to the 2021 China Statistical Yearbook, 10.62 million babies were born in China in 2021 (National Burea of Statistics of China, 2021). Thus, there is an urgent need for innovative and accessible interventions to alleviate pregnancy stress, and MBCP courses localized in China provide Chinese women with a new option to alleviate pregnancy stress and improve their mental health. Our study provides a theoretical reference for promoting and applying localized MBCP courses for pregnant women in China.

### 4.1. Limitations

This study has the following limitations. First, the participants were primarily pregnant women with a high level of education. In the intervention group, 74.47% of participants held a bachelor’s degree or higher, compared to 66.65% in the control group. We hypothesized that the effect of the mindfulness course may be related to the level of education of the participants; those with a higher level of education may comprehend the course material better and, therefore, be more responsive to the intervention. We are unsure whether the “2-day onsite and 21-day online” simplified version of the MBCP course would have the same effect if the participants were pregnant women with a lower level of education. Second, the inclusion criteria were limited to primiparas. Pregnancy stress levels have been found to be higher in primiparas (Wheeler et al., 2018), and those with higher stress may be more sensitive to mindfulness interventions. We are unsure whether the simplified version of the MBCP course would have the same effect for nonprimiparas. These two aspects of the study limit the extrapolation of the results. Third, the sample size of this study was relatively small, and the differences in the magnitude of changes in some dimensions of stress levels and mindfulness levels between the two groups after the intervention were not well demonstrated.

## 5. Conclusion

Our findings indicate that mindfulness training during pregnancy can effectively relieve stress and improve the physiological stress response function of the HPA axis in pregnant women. The “2-day on-site and 21-day online” simplified version of the MBCP course localized for pregnant women in China appears to be an acceptable and effective intervention for maternal mental health. However, health economics must still determine whether the program can be widely promoted among pregnant women in China.

## Data availability statement

The raw data supporting the conclusions of this article will be made available by the authors, without undue reservation.

## Ethics statement

The studies involving human participants were reviewed and approved by Ethics Review Committee of the National Center for Women and Children (Chinese Center for Disease Control and Prevention, Beijing, China; approval no. FY2020-10). The patients/participants provided their written informed consent to participate in this study.

## Author contributions

RZ and FS conceived the research. SW, MS, and CZ designed the survey. RZ, SW, MS, CZ, DZ, JW, YL, and KL conducted the survey. SW and CZ conducted the statistical analyses. SW wrote the primary draft and prepared advanced drafts for publication. SW, CZ, and MS finalized the report. TX and XP supervised the writing of the report. All authors contributed to the article and approved the submitted version.

## Funding

This study was funded by the National Center for Women and Children’s Health of the Chinese Center for Disease Control and Prevention.

## Conflict of interest

The authors declare that the research was conducted in the absence of any commercial or financial relationships that could be construed as a potential conflict of interest.

## Publisher’s note

All claims expressed in this article are solely those of the authors and do not necessarily represent those of their affiliated organizations, or those of the publisher, the editors and the reviewers. Any product that may be evaluated in this article, or claim that may be made by its manufacturer, is not guaranteed or endorsed by the publisher.
